# Reduced Graphene Oxide/Carbon Nanotube Composites as Electrochemical Energy Storage Electrode Applications

**DOI:** 10.1186/s11671-018-2582-6

**Published:** 2018-06-15

**Authors:** Wenyao Yang, Yan Chen, Jingfeng Wang, Tianjun Peng, Jianhua Xu, Bangchao Yang, Ke Tang

**Affiliations:** 1 Post-doctoral Research Station, College of Material Science and Engineering, Chongqing University, Chongqing, 400044 People’s Republic of China; 2 Chongqing Key Laboratory of Micro/Nano Materials Engineering and Technology, Chongqing University of Arts and Sciences, Chongqing, 402160 People’s Republic of China; 3 Sichuan Province Key Laboratory of Information Materials and Devices Application, College of Optoelectronic Technology, Chengdu University of Information Technology, Chengdu, 610225 People’s Republic of China; 4 Chongqing Engineering Research Center of New Energy Storage Devices and Applications, Chongqing University of Arts and Sciences, Chongqing, 402160 People’s Republic of China; 5 College of Opto-electronic Information, University of Electronic Science and Technology, Chengdu, 610054 People’s Republic of China

**Keywords:** Reduced graphene oxide, Carbon nanotube, Specific capacitance, Nanocomposite material, Electrochemical reduction

## Abstract

We demonstrate an electrochemical reduction method to reduce graphene oxide (GO) to electrochemically reduced graphene oxide (ERGO) with the assistance of carbon nanotubes (CNTs). The faster and more efficient reduction of GO can be achieved after proper addition of CNTs into GO during the reduction process. This nanotube/nanosheet composite was deposited on electrode as active material for electrochemical energy storage applications. It has been found that the specific capacitance of the composite film was strongly affected by the mass ratio of GO/CNTs and the scanning ratio of cyclic voltammetry. The obtained ERGO/CNT composite electrode exhibited a 279.4 F/g-specific capacitance and showed good cycle rate performance with the evidence that the specific capacitance maintained above 90% after 6000 cycles. The synergistic effect between ERGO and CNTs as well as crossing over of CNTs into ERGO is attributed to the high electrochemical performance of composite electrode.

## Background

In the last decades, supercapacitors have been widely studied in order to meet the rapidly growing demands of new energy-device with high-power, high energy, high charge/discharge rates and long cyclic life [1]. Generally, activated carbon, carbon nanotubes, mesoporous carbon, nano-carbon have been investigated for use as electrodes in electrochemical double-layer supercapacitors. Besides, the pseudo-supercapacitors materials, conductive polymers and transition metal oxides, storing energy through a faradic process have been widely explored [2, 3]. Recently, graphene and its composites have attracted a wide range of research for the electrode material because of their large surface area, high carrier mobility, and excellent electrochemical stability [4–6]. As a one-atom thick layer of carbon atoms arranged in a honey-comb lattice, graphene is well-known for its high specific capacitance as energy storage applications [7, 8]. However, large area preparation of high quality graphene films as energy storage applications are still in challenges [9, 10].

As for preparation methods, mechanical exfoliation by sonication [11, 12], epitaxial growth on metal or silicon carbide [13, 14], chemical vapour deposition [15–17], and etc have been investigated extensively. Among these methods, the electrochemical reduction of graphene oxide (GO) has aroused great research interest in recent years due to its advantages, such as relatively simple, economic, manageable and eco-friendly [18–22]. However, the obtained pristine electrochemically reduced graphene oxide (ERGO) exhibits low specific capacitance result from their characteristic of easy agglomerate.

## Presentation of the hypothesis

Herein, some tentative works have been demonstrated to prepare highly opened reduced graphene oxide (RGO) structure incorporated with other nanostructures, such as nanoparticles [23, 24], nanotubes [25] and nanowires [26, 27]. The well interaction between the components would result in good synergistic effect in these nanocomposites, which leads to excellent electrical and electrochemical performance. However, well dispersion of these nanostructures into RGO is still challenged due to the feasible preparing method and bad interaction between various components.

## Testing the hypothesis

Here, we demonstrate an in situ electrochemical method to prepare high performance ERGO composites. The high conductive carbon nanotubes (CNTs) was added into GO sheets during the reduction process of GO.

## Implications of the hypothesis

Due to the entangled framework of CNTs, it could be beneficial to reduce agglomerate of the GO sheets and the obtained ERGO shows more highly opened structures. Moreover, the excellent conductivity of CNTs would also beneficial to reduce the GO into ERGO with fast speed and more efficiently. With finely controlling of addition ratio, the obtained ERGO/CNTs nanocomposites show excellent electrical and electrochemical performance, which shows promising future as electrochemical energy storage electrodes.

## Methods

### Synthesis of ERGO/CNTs

The GO was prepared from natural graphite flakes by modified Hummer’s method. An aqueous GO dispersion solution about 1.5 mg/ml was used to prepare composites, and the size of the GO sheet is controlled with less than 650 nm. The graphite flakes (XF055 7782-42-5) and a water solution of CNT (XFWDST01 1333-86-4) dispersion were also purchased from Nanjing XFNANO Materials Tech., Co., Ltd. All materials were used as received.

For the preparation of composite GO/CNT solution, the GO solution was firstly ultrasonicated in an electric-heated thermostatic water bath for 20 min at 40 °C. Then, CNT solution purchased from XFNANO was added into GO solution with the different mass ratios and continued to stir for 15 min. Subsequently, a spray-coating method was used to deposit GO and CNTs onto ITO substrate, and then, the substrate was treated in a vacuum oven at 60 °C for 2 h. Finally, the obtained GO/CNT films were put into an electrolytic tank, the GO was electrochemically reduced into ERGO, and an ERGO/CNT composite film was obtained. Electrochemical reduction of as-prepared GO/CNT films were carried out in a three-electrode system (CHI660D electro-chemical workstation, Chenhua, Shanghai, China) with a 0.5 M (pH 6.0) Na_2_SO_4_ aqueous solution as working electrolyte. A platinum disk and an Ag/AgCl electrode were used as the counter and reference electrode, respectively.

### Characterization

The electrical conductivity of ERGO and ERGO/CNTs was characterized by a four-probe testing system with a SZ-100 model (Baishen, Suzhou, China). FT-IR spectrum was characterized with an ALPHA analysis instrument (Germany). Surface morphologies of ERGO and the composite films were characterized by scanning electron microscopy (SEM) with a model Philips XL30-FEG. X-ray diffraction system (XRD, X’Pert Pro MPD DY129) and Raman spectroscopy (Advantage 633 nm) were used to investigate the crystallographic structure of the films. The cyclic voltammetry (CV) and galvanostatic charge/discharge curves (GCD) of composite films were also obtained from CHI660D electro-chemical workstation (Chenhua, Shanghai, China).

## Results and Discussion

**Fig. 1:**
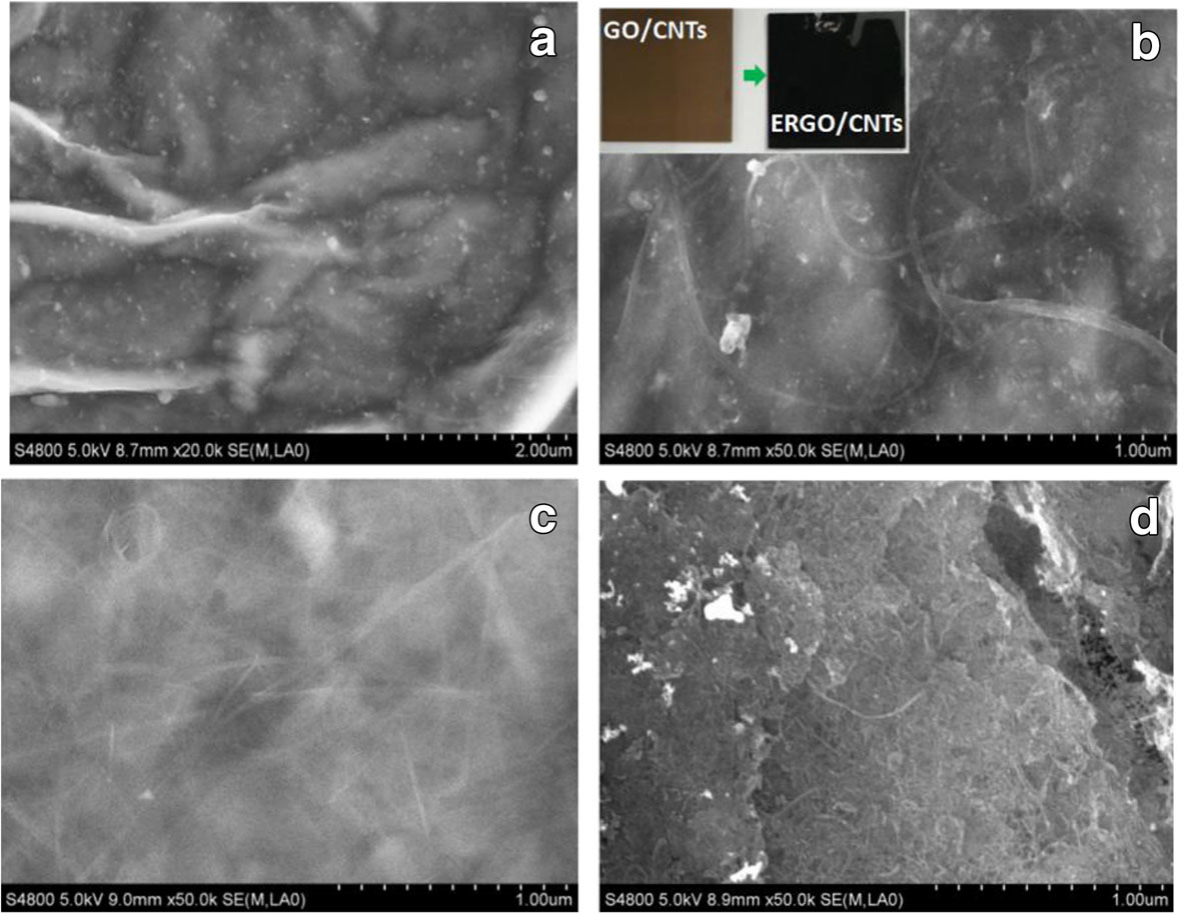
SEM images of ERGO (**a**) and ERGO/CNTs obtained from GO/CNTs with different mass ratios: **b** GO/CNTs = 100:1, **c** GO/CNTs = 50:1, and **d** GO/CNTs = 10:1; the inset images in (**b**) are photo pictures of GO/CNTs before and after electrochemical reduction

The purpose about addition of CNTs into GO sheets is that the CNTs cannot only supply the entangled structure after the physical mixing to avoid the agglomerating of GO sheets but also improve the conductivity of composite GO/CNT films for fast reduction of GO into ERGO. Figure 1 shows the SEM images of ERGO and ERGO/CNT films electrochemically reduced from GO/CNT films with a different GO/CNT mass ratio. The obvious color change of composite films from faint yellow to deep black (as shown in the inset of Fig. 1b) indicates the successful reduction of GO into ERGO. From Fig. 1a, b we can see the entangled structure of CNTs and the ridge-like wrinkle structure of ERGO are exhibited in composite films. The CNTs have embedded in ERGO evenly after the physical mixing and electrochemical reduction. The embedded CNTs can protect the ERGO sheets from agglomerating effectively, and this wrinkled and entangled structure is able to offer higher surface area than pristine ERGO. This highly opened composite nanostructure is suitable for easily and sufficiently adsorbing electrolyte ions onto electrode surface during electric double-layer energy storage. Furthermore, with the increasing mass ratio of CNTs (as shown in Fig. 1c, d), more CNTs penetrate into ERGO as a support and an aggregated structure of CNTs are presented.

**Fig. 2:**
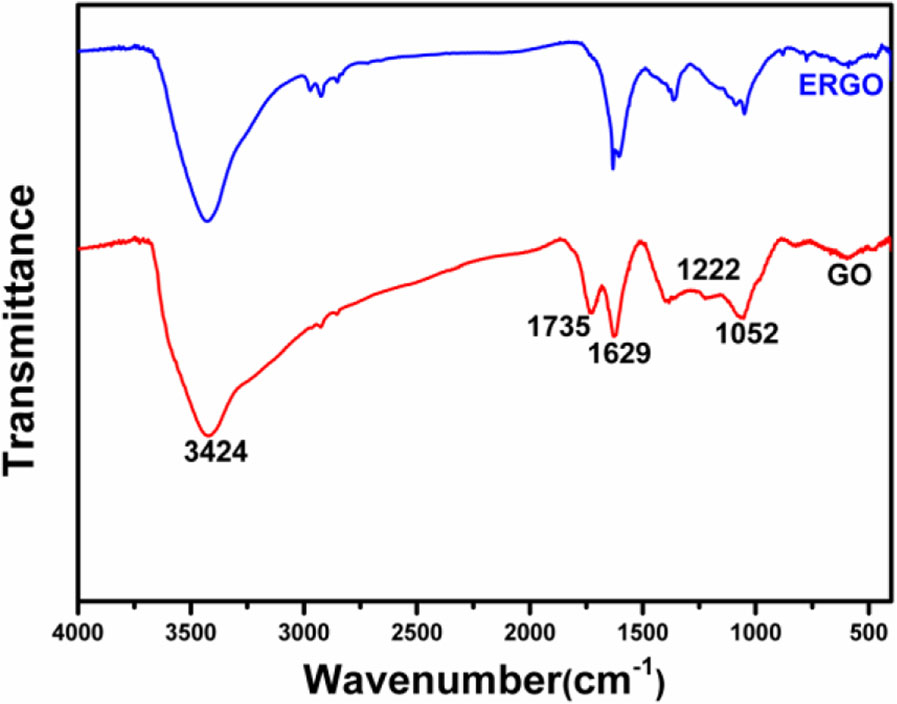
FT-IR spectrum of GO and ERGO

The functional groups of GO and ERGO are characterized by FT-IR spectrum, which are shown in Fig. 2. As for graphene oxide, the peak at 3424 cm^−1^ is ascribed to O–H stretch. The peaks at 1735 and 1629 cm^−1^ are a result from C=O stretch and aromatic C=C, respectively. The peak at 1222 cm^−1^ rises from O–H bending and the peak at 1052 cm^−1^ are ascribed to epoxy C–O stretch and alkoxy C–O stretch. These identified function groups by FT-IR spectrum indicates the oxygen-containing nature of GO. After an electrochemical reduction, the obviously weakened peaks in spectrum are 1735 and 1222 cm^−1^ [28], indicating the well elimination of these oxygen-containing groups.

The reduction process of GO is also confirmed by the change of conductivity of nanocomposites, as shown in Table 1. It can be seen that, firstly, the addition of CNTs into GO sheet has improved the electrical ability of GO/CNTs composites. With the addition ratio of GO to CNTs from 0 to 50:1 and 10:1, the electrical resistance of nanocomposite varies from a MΩ/sq. to a kΩ/sq. level. After the electrochemical reduction, an obvious enhancement of electrical conductivity is achieved in nanocomposite, indicating effectively reducing of GO into ERGO. The remarkable improvement in electrical conductivity of ERGO film is attributed to the elimination of the oxygen functionalities during the electrochemical reduction, and the symmetrical sp^2^ C=C bonds are rebuild for better transferring of carriers [20]. Accordingly, with the increasing of electrical conductivity, a more continuous and complete conducting path is formed in ERGO/CNT composite. The results in Table 1 also reveal that, after the electrochemical reduction, there is no distinct conductivity difference found between the ERGO and ERGO/CNTs nanocomposite, and this results indicate that the reduced ERGO exhibits a comparable electrical conducting ability with CNTs.

**Table 1:** Electrical conductivity of GO and GO/CNTs with different mass ratios before and after electrochemical reduction

Film samples	GO	GO/CNTs (50:1)	GO/CNTs (10:1)
Conductivity before reduction	23.5 MΩ/sq	79.8 kΩ/sq	47.8 kΩ/sq
Conductivity after reduction	11.17 kΩ/sq	8.35 kΩ/sq	9.27 kΩ/sq

**Fig. 3:**
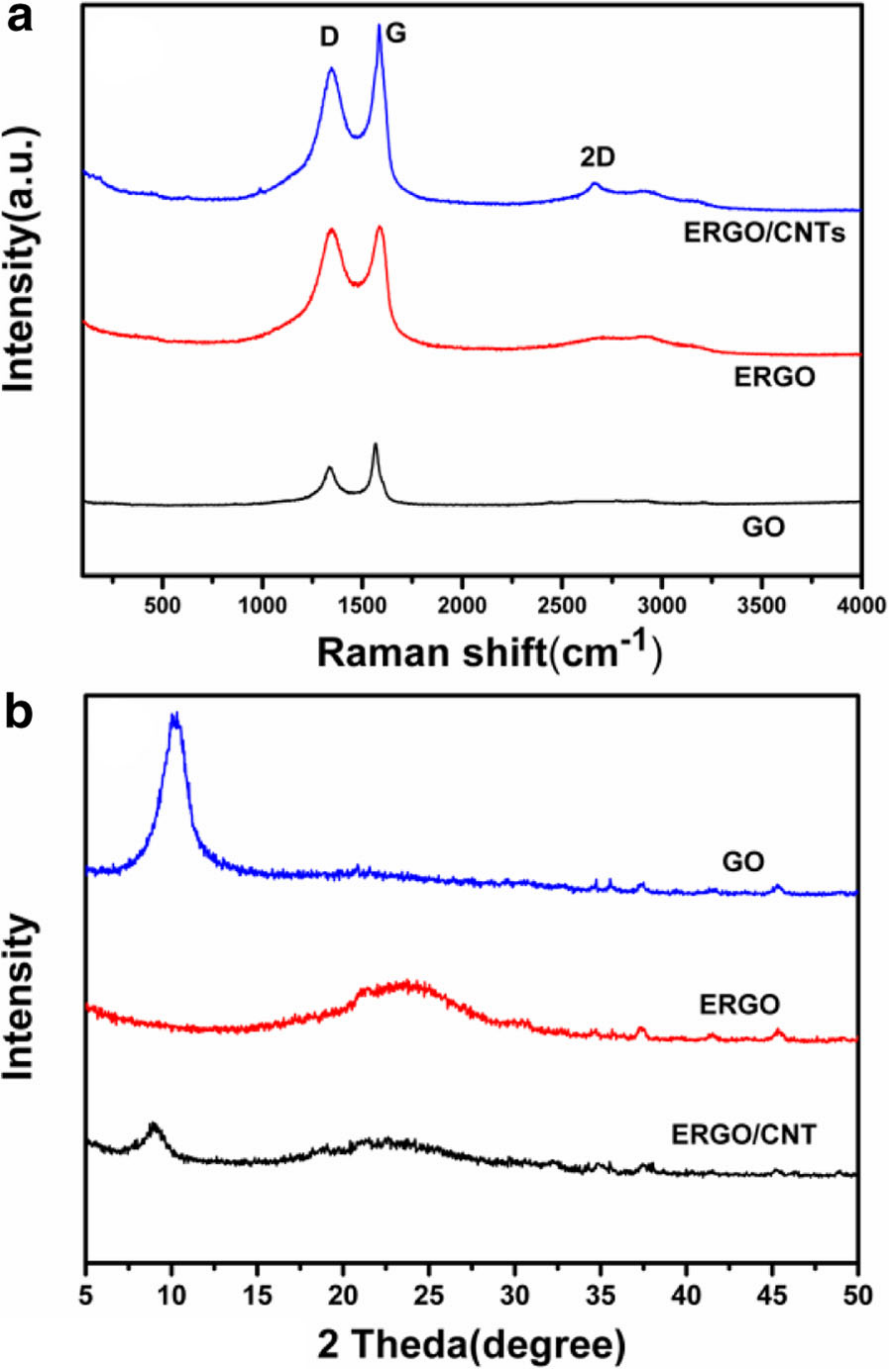
**a** Raman spectra and **b** X-ray diffraction spectra of the pristine GO, ERGO, and ERGO/CNTs

The structure change of GO after the electrochemical reduction is characterized by Raman spectra and X-ray diffraction analysis, which are shown in Fig. 3. From Fig. 3a, a D band at ~ 1345 cm^−1^ and a G band at 1583 cm^−1^, which are assigned to the disordered structural defects and the *E*_2g_ phonon of sp2 carbon atoms [29], are presented, respectively. It is noted that the ERGO film exhibits a higher *I*_D_/*I*_G_ ratio than the pristine GO film due to the decreased amount of defects after electrochemical reduction. The ERGO/CNT composite shows a lower *I*_D_/*I*_G_ ratio than ERGO due to the addition of CNTs.

XRD patterns of GO, ERGO, and ERGO/CNTs also reveal the electrochemical reduction of GO into ERGO after the mixing with CNTs (as shown in Fig. 3b). As for GO, an additional peak at 10.3° is observed, which is attributed to the (001) diffraction peak of GO. The larger interlayer distance of GO nanosheets might be due to the existence of oxygen-containing functional groups on the sheet surface [30]. After the electrochemical reduction, the (001) diffraction peak of GO disappears and a broad diffraction peak (002) approximately 24.3° appears. The interlayer spacing of ERGO was 0.39 nm, slightly larger than that of graphite, which was resulted from the small amount of residual oxygen-containing functional groups or other structural defects. Besides the feeble and broad diffraction peak (002), the ERGO/CNTs also show a weak diffraction peak at 8.4°. We conclude that comes from the mixing of GO with CNTs, and this mixing structure leads to the shift of diffraction peak after electrochemical reduction of GO.

**Fig. 4:**
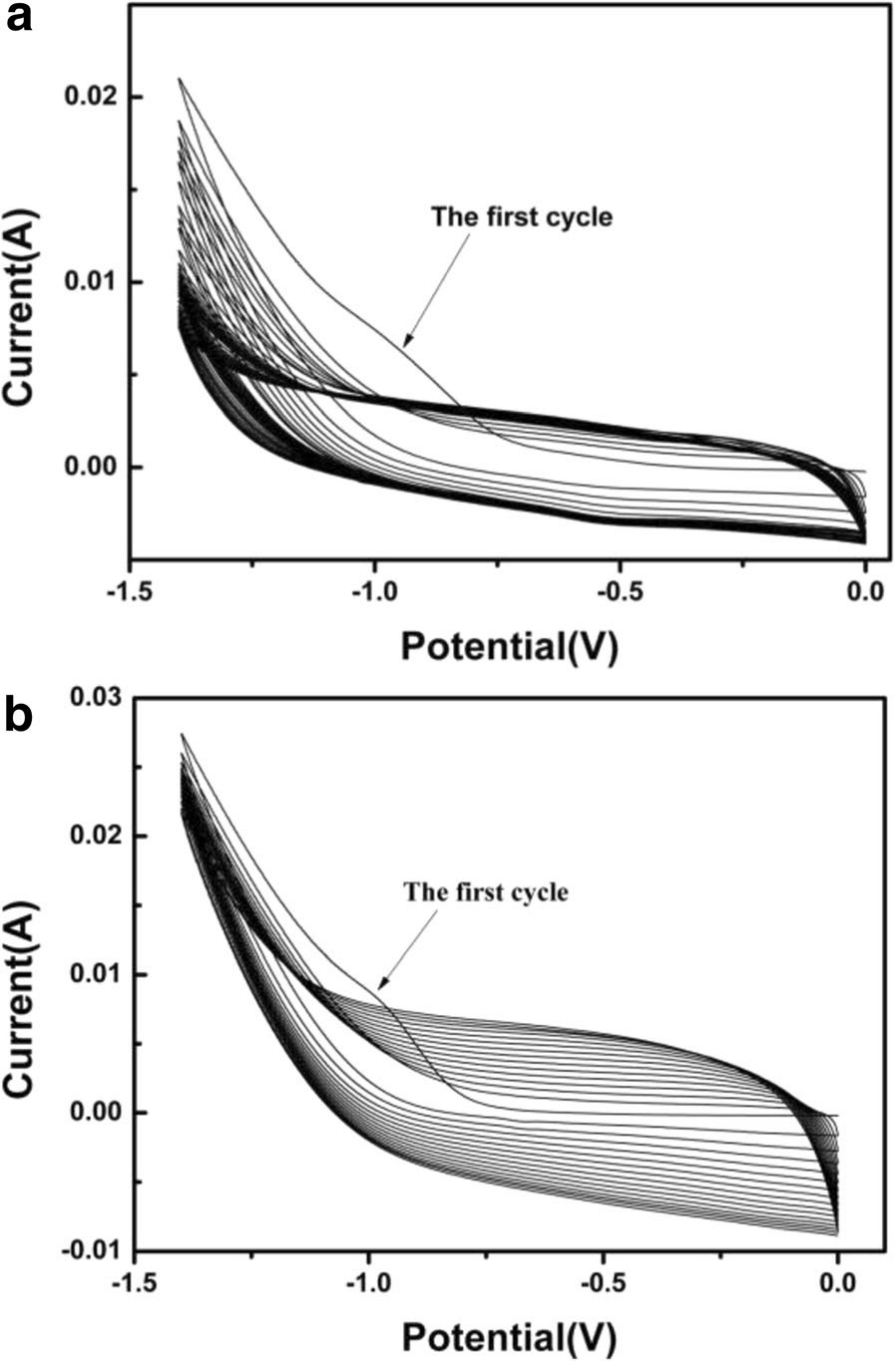
Electrochemical reduction of **a** GO and **b** GO/CNTs (mass ratio 50:1) in 0.5 M Na_2_SO_4_ (pH 6.0) at a scan rate of 50 mV/s

The electrochemical reduction process of GO and GO/CNTs are characterized by CV curves (as shown in Fig. 4). Both GO film and the GO/CNTs film are electrochemically reduced at the potential range from 0 to − 1.4 V in a 0.5 M Na_2_SO_4_ electrolyte solution (pH 6.0). Obviously, the cathodic peak appeared at about − 0.75 V during the first cycle results from the partial elimination of the major functional groups on the surface of GO sheets, such as epoxy, carboxyl, and hydroxyl [20]. It should be noted that, compared with the pristine GO film, the reduction process of the composite GO/CNT films is more rapid with the evidence that the GO/CNT film shows larger reaction current at the first cycle. Moreover, the GO/CNT films come to stable current with less reaction cycles, which means that the reducing process of GO in GO/CNTs is faster than that of pure GO at the same electrochemical conditions. We conclude that the high conductivity of CNTs improves the electrical ability of GO/CNTs, and the enhanced conductivity results in faster electron transferring between the electrode and GO/CNTs, leading to a faster reduction process of GO into ERGO.

**Fig. 5:**
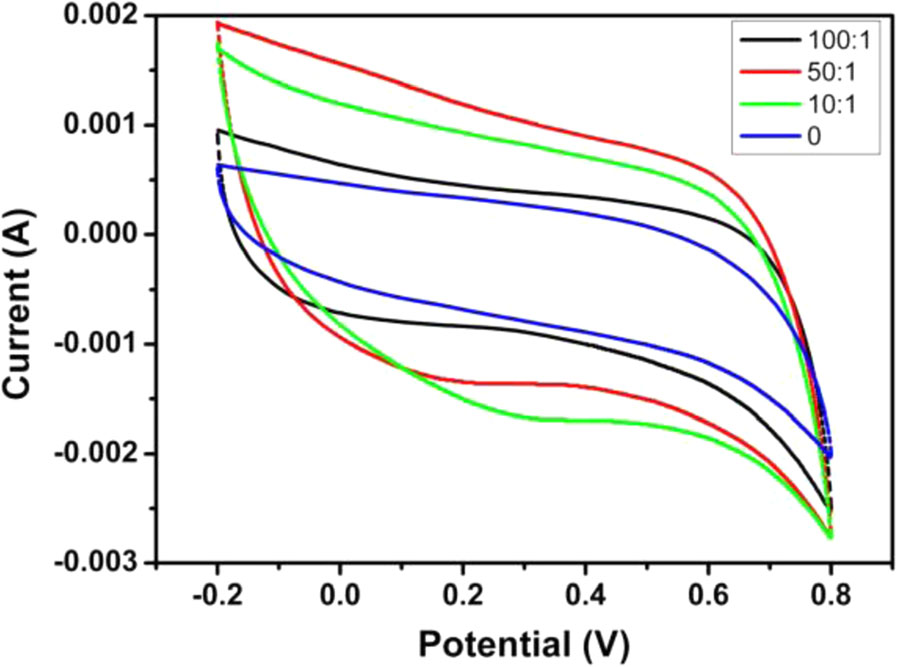
Cycle voltammetry curves of ERGO and ERGO/CNTs with a different GO/CNTs mass ratio at 50 mV/s. (All films were prepared by CV at a scan rate of 50 mV/s)

Figure 5 shows cycle voltammetry curves of ERGO and ERGO/CNTs electrochemically reduced from GO/CNTs with different mass ratios at 50 mV/s. All the films were prepared by CV method at a scan rate of 50 mV/s. The results reveal that the mixing ratio of GO and CNTs in composites takes a great effect on specific capacitance of composite electrodes. The incorporation of high surface-to-volume ratio CNTs improves the energy storage density of electrode greatly. Table 2 shows the calculated specific capacitance of different electrodes. From Table 2, we can see the specific capacitance of composite electrode dramatically increases from 156.3 to 279.4 F/g with the increasing of CNT/GO mass ratio, which is reasonable for the surface area increasing of composite electrode is due to the appropriate addition of CNTs and corresponding benefits of reducing agglomerate of GO sheets. The CNT-enriched composite electrode exhibits obviously larger specific capacitance than pure ERGO, and these specific capacitance results are well consistent with the result of CV testing. However, compared with the 50:1 and 10:1 mass ratios, the further increasing of CNTs does not obviously increase the specific capacitance accordingly, and a reduced specific capacitance of electrode is observed. We conclude that high mass ratio of CNTs in composite structure results in an unpredicted agglomeration of CNTs, which leads to the inadequate interaction of CNTs with ERGO sheets for further improving the efficient surface area of composite. So, controlling the distribution and loading of CNTs in ERGO matrix during the electrochemical reduction process is very important to optimize composite electrode with high specific capacitance.

**Table 2:** Calculated specific capacitance of different electrodes (F/g)

Samples (mass ratio of GO to CNTs)	Non-CNTs	100:1	50:1	10:1
Specific capacitance	156.3	178.1	279.4	203.5

**Fig. 6:**
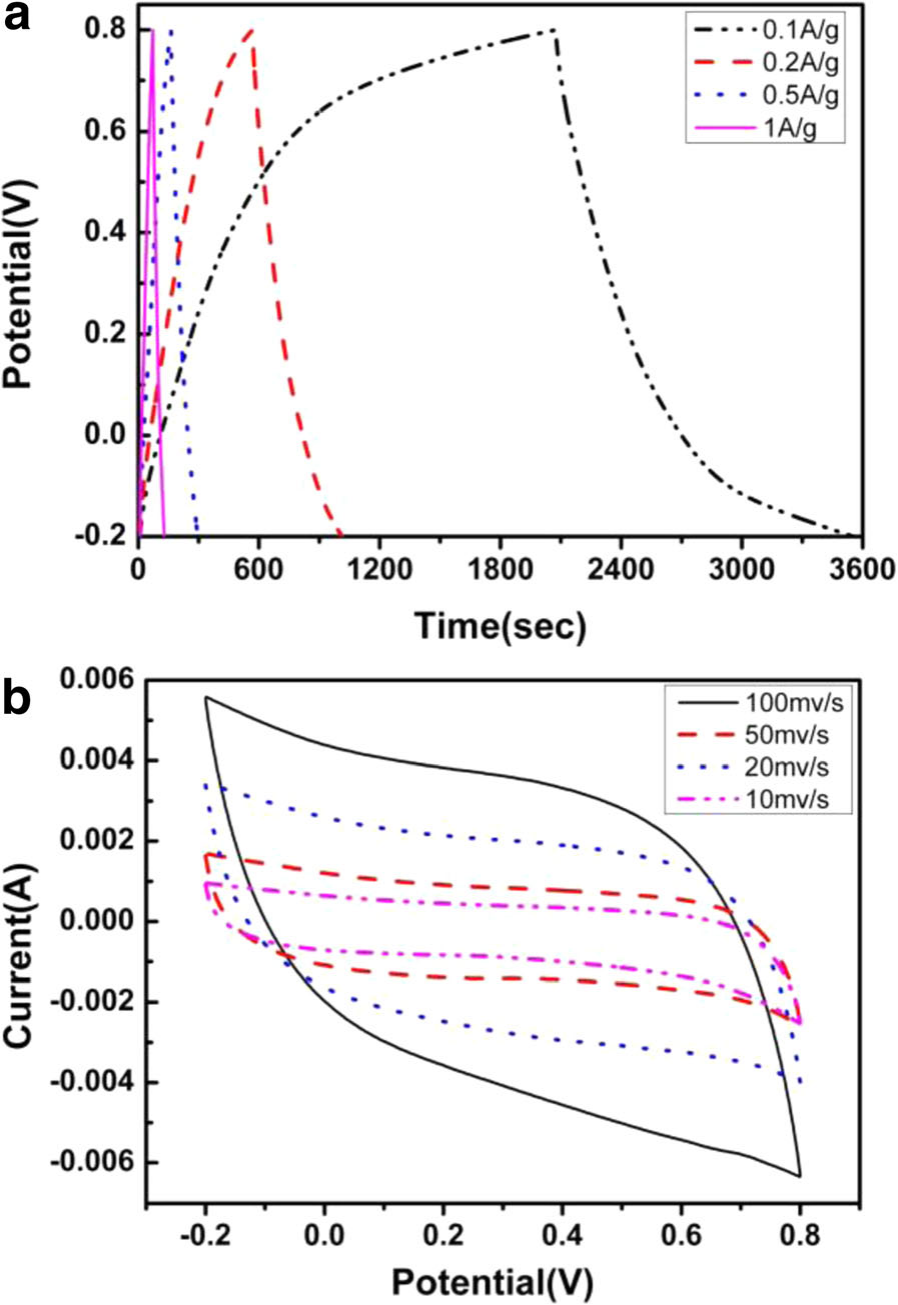
Galvanostatic charge/discharge curves (**a**) and CV pattern (**b**) of the as-prepared film in 0.5 M Na_2_SO_4_ (pH 6.0) (mass ratio of 50:1 and scan rate of 50 mV/s)

It is well known that high-rate capability is a key index for electrochemical capacitor electrodes. Rate performance of ERGO/CNT composite electrodes is shown in Fig. 6a. The specific capacitance of all composite electrodes shows a decreasing tendency with the increase of current due to the fact that the accessibility of electrolyte ions to active sites of the electrode is limited at higher current density [20]. The uniform distribution of CNTs into ERGO nanosheets can effectively improve the rate capability compared with pure ERGO electrode with agglomerate structure. As shown in Fig. 6a, the ERGO/CNT electrode shows the excellent specific capacitance at a current density of 1 A/g. This means that the highly opened composite electrode can not only afford high specific capacitance but also keep high capacitance retention at high current density. The uniform distribution of CNTs into ERGO sheets is reasonable for the high rate performance of composite electrodes. The CV curves of ERGO/CNT films (Fig. 6b) exhibit almost the rectangular-like shape with increasing scanning voltage, indicating a remarkable capacitive behavior and excellent reversibility of their charge/discharge process.

**Fig. 7:**
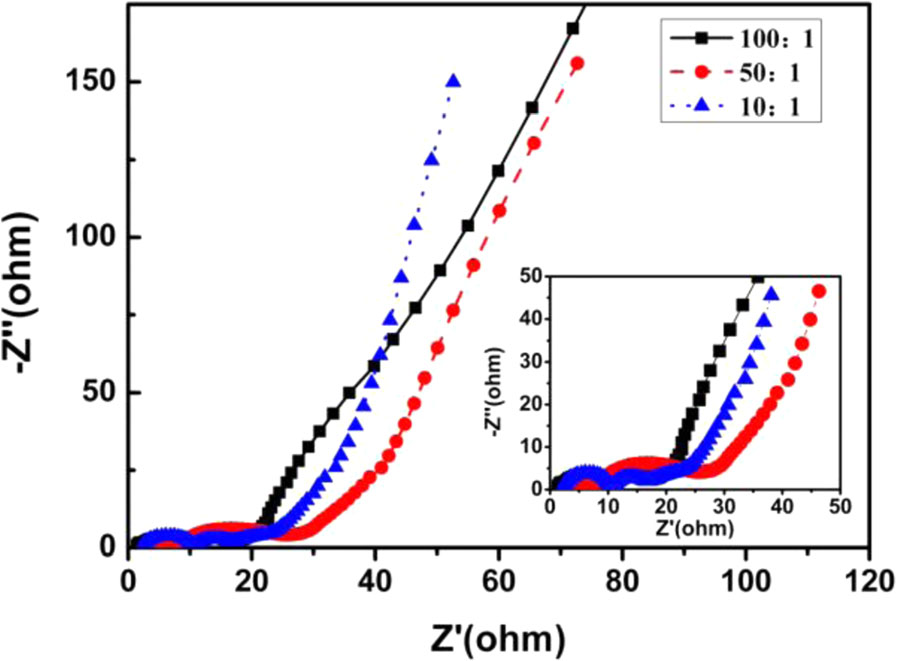
Nyquist plots of different composite electrodes

Figure 7 is Nyquist plots of different composite electrodes. It can be seen that the composite electrodes show almost the same inner resistant (Rs) with pure ERGO electrode, which is lower than GO electrode. The composite electrode loading on CNTs shows no obvious influence on electrode Rs, indicating the comparable conductive performance of ERGO and CNTs. However, an obvious decrease of specific capacitance is observed with the increasing of GO/CNT mass ratio to 10:1, as shown in Fig. 5 and Table 2. Consequently, the excellent resistance and specific capacitance performance of composite electrode is reasonable and depending on the optimized loading and distribution of CNTs in ERGO sheets.

**Fig. 8:**
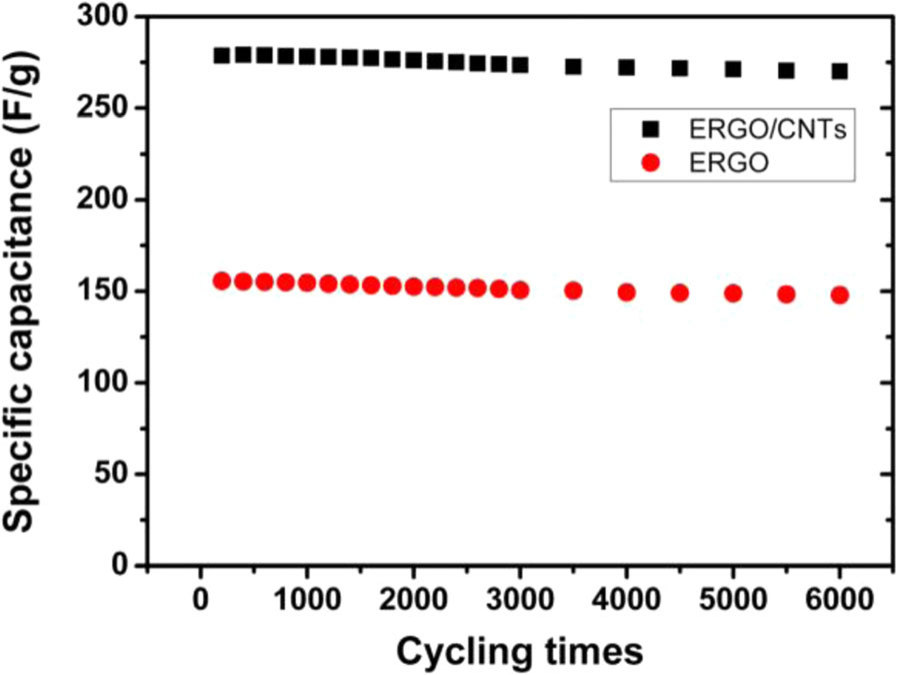
Cycling rate performance of different electrodes at 1.2 A/g scan current density

Cycle rate performance of electrode films is also a vital factor for the practical application in electrochemical capacitor. As shown in Fig. 8, the rate performance of ERGO/CNTs (obtained from GO/CNT mass ratio = 50:1) and pure ERGO is evaluated by charging/discharging at the same current densities. For ERGO/CNT electrode, the specific capacitance maintained above 90% after 6000 cycles at a 1.2 A/g scan current density. The results indicate a good cycling ability of this nanotube/nanosheet composite electrode. The penetration of CNTs into ERGO provides a robust support for electrochemical activity of ERGO. Therefore, the alternating nanotube/nanosheet structure affords excellent mechanical strength for the long-term cycling of charge/discharge. It also can be seen in Fig. 8 that the pure ERGO electrode also presents good cycling ability only with lower specific capacitance, which results from stable EDLC and agglomerate structure of ERGO. So, it is crucial and valuable to build highly opened and stable carbon nanomaterial structure to obtain high-performance electrochemical energy storage electrode with large specific capacitance and high stability.

## Conclusions

In conclusion, we utilized a simple electrochemical method to prepare an ERGO/CNT composite films, and the pre-added CNTs into GO sheets play an important role as a reduction accelerant. High-efficiency reduction of GO was obtained, and the obtained ERGO/CNT composite film showed excellent electrochemical performance. At a mass ratio of 50:1 and the scan rate of 50 mV/s, the composite film exhibited a high specific capacitance about 279.4 F/g and showed excellent reversibility. Furthermore, this simple and versatile synthesis technique for providing graphene-based materials shows promising future in various applications such as assembly of electrochemical capacitors.

## Abbreviations

CNTs: Carbon nanotubes
CV: Cyclic voltammetry
ERGO: Electrochemically reduced graphene oxide
GCD: Charge/discharge curves
GO: Graphene oxide
XRD: X-ray diffraction system
